# Glucocorticoids and HPA axis regulation in the stress–obesity connection: A comprehensive overview of biological, physiological and behavioural dimensions

**DOI:** 10.1111/cob.12725

**Published:** 2024-12-02

**Authors:** Robin Lengton, Myrte Schoenmakers, Brenda W. J. H. Penninx, Mariëtte R. Boon, Elisabeth F. C. van Rossum

**Affiliations:** ^1^ Department of Internal Medicine, Division of Endocrinology, Erasmus MC University Medical Center Rotterdam Rotterdam The Netherlands; ^2^ Obesity Center CGG, Erasmus MC University Medical Center Rotterdam Rotterdam The Netherlands; ^3^ Department of Biological Psychology Vrije Universiteit Amsterdam The Netherlands; ^4^ Amsterdam Public Health research institute Amsterdam UMC Amsterdam The Netherlands; ^5^ Department of Psychiatry and Amsterdam Public Health, Amsterdam UMC Vrije Universiteit Amsterdam The Netherlands

**Keywords:** Obesity, physiological, stress, stress domains, stress systems

## Abstract

Chronic stress, characterized by increased long‐term exposure to the glucocorticoid hormone cortisol, is increasingly linked to obesity development. Still, various knowledge gaps persist, including on underlying pathophysiological mechanisms. The aim of the current review is to provide the latest insights on the connection between stress and obesity. We discuss three biological stress systems—the autonomic nervous system, the hypothalamus–pituitary–adrenal (HPA) axis and the immune system—and their link with obesity, with a particular focus on the HPA axis. The role of cortisol and its regulatory variations (including glucocorticoid rhythmicity and altered sensitivity) in adipose tissue biology and obesity development is discussed. Moreover, we highlight the physiological, affective, cognitive and behavioural dimensions of the stress response offering a deeper understanding of how stress contributes to obesity development and vice versa. Finally, stress as a treatment target for obesity is discussed. We conclude that the link between stress and obesity is complex and multifaceted, influenced by physiological, affective, cognitive and behavioural stress response mechanisms, which especially when chronically present, play a key role in the development of obesity and associated cardiometabolic diseases. This necessitates integrated approaches tailored to individual needs, including lifestyle modifications, behavioural interventions, psychosocial support and possible additional pharmacological interventions.

## 1 INTRODUCTION

Stress and obesity are the two interrelated health concerns that have garnered substantial attention in recent years due to their high prevalence and impact on public health. Over short periods of time, stress responses can be beneficial by focusing attention, mobilizing energy and motivating adaptive behaviour. When stress occurs frequently and/or becomes chronic, it can contribute to the development of both mental (e.g., depression, anxiety, burnout) and cardiometabolic (e.g., type 2 diabetes, cardiovascular disease) diseases. Of note, obesity is a significant factor that has been linked to both mental health disorders and cardiometabolic diseases, reflecting its complex role in these health outcomes.^1^, ^2^ In the past years, there has been increasing evidence for a relation between chronic stress, primarily through increased exposure to the glucocorticoid (GC) stress hormone cortisol and the development of obesity.^3^ Cortisol is known for its role in regulation of the stress response, energy metabolism, appetite and adipose tissue distribution,^4^ making it a central player in the intricate link between stress and obesity. However, understanding the link between stress and obesity also requires consideration of other physiological systems involved in the body's response to stress, namely, the autonomic nervous system (ANS) and the immune system. ANS dysregulation, often indicated by overactivation of the sympathetic nervous system (SNS), has been linked to the development of metabolic syndrome components.^5^, ^6^ On the other hand, chronic stress can also contribute to immune cell dysfunction and an increased risk of various diseases, including obesity.^3^, ^7^

This review will summarize the current knowledge and recent novel insights on the relation and underlying pathophysiological mechanisms between stress and obesity, with a focus on the hypothalamic–pituitary–adrenal (HPA) axis as a component of the physiological stress response, which also involves the ANS and the immune system. Beyond these physiological aspects, this review will also explore the affective, cognitive and behavioural domains of the stress response in relation to obesity. More specifically, stress can influence emotional well‐being, cognitive functions and behavioural patterns related to eating, physical activity and sleep. Understanding these four domains, and their interrelations, provides a holistic perspective on how stress contributes to obesity development. Furthermore, we will also discuss stress as a treatment target for obesity.

## 2 THE STRESS SYSTEM

**FIGURE 1:**
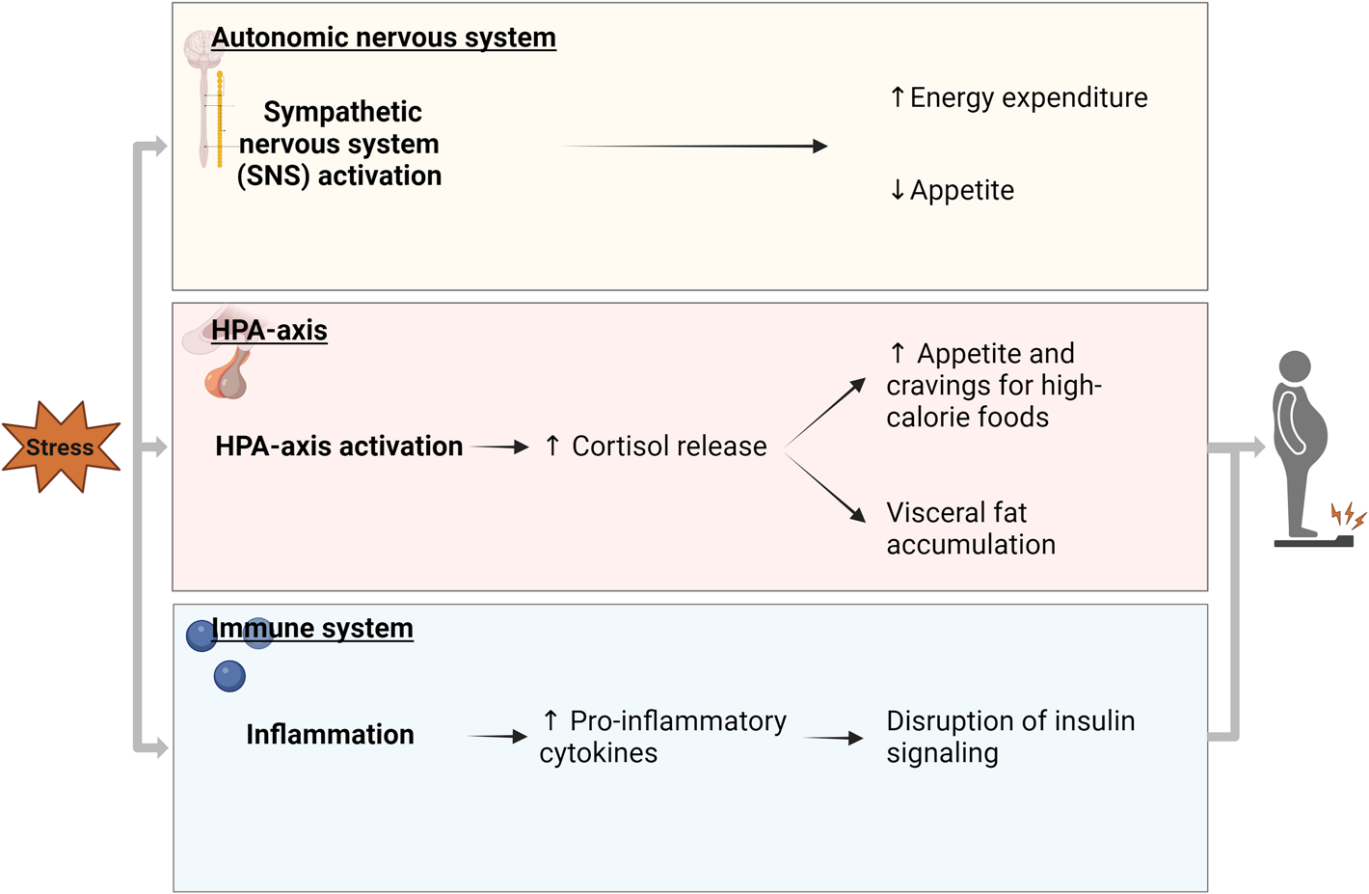
The stress system in relation to obesity. To respond to the stressors, a highly sophisticated stress system has been developed. This stress system consists of three distinct but interrelated physiological systems: the autonomic nervous system, the hypothalamic–pituitary–adrenal (HPA) axis and the immune system. *Source*: Created with BioRender.com.

**FIGURE 2:**
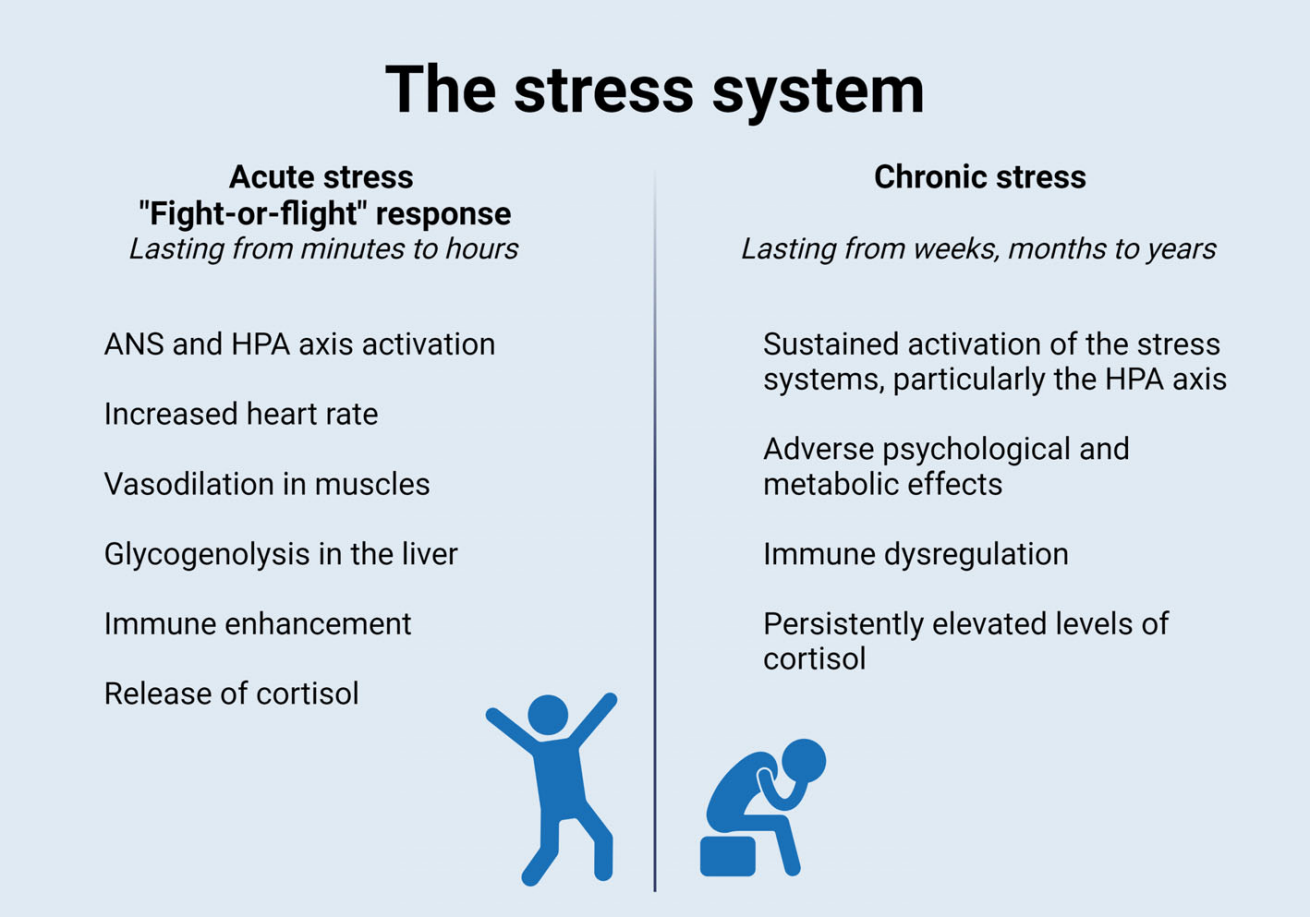
Effect of acute and chronic stress on the body. This figure illustrates the physiological responses of acute and chronic stress on the body. *Source*: Created with BioRender.com.

Stress is a person–environment interaction that results in physical or psychological tension and impacts all aspects of daily life.^8^ Potential stressors that can evoke a stress response, can be external or internal.^8^ External stressors refer to factors in the environment outside the person and internal stressors to factors in the mind (e.g., anticipation, memories) or body (e.g. pain) of a person.^8^ To respond to these stressors, a highly sophisticated stress system has been developed in our bodies. This stress system consists of three distinct but interrelated physiological systems: the ANS, the HPA axis, and the immune system (Figure 1). These systems provide the appropriate central and peripheral neuroendocrine responses to stressors.^9^ The duration of the stressor affects which of these physiological systems is activated. Accordingly, stress can be categorized into two distinct forms: acute stress and chronic stress (Figure 2). Apart from their duration, these forms of stress differ significantly in their causes, physiological responses and long‐term impact.

### 2.1 Acute stress

Acute stress is the body's immediate reaction to perceived threats or challenges. It is often referred to as the “fight‐or‐flight” response, lasting from minutes to hours. When faced with an acute stressor, the sympathetic division of the ANS releases catecholamines such as epinephrine and norepinephrine from the medulla (centre) of the adrenal gland, both mediating the fight‐or‐flight response, as well as stimulates release of catecholamines by the brain. This coincides with physiological changes to bring the body into fight mode, including increased heart rate and vasodilation in muscles.^10^ In particular, epinephrine stimulates glycogenolysis in the liver providing energy to fuel defensive responses.^10^ Although peripherally produced epinephrine and norepinephrine cannot cross the blood–brain barrier, the locus coeruleus within the brain also produces norepinephrine upon acute stress to mimic the peripheral actions.^11^ Its role is to support arousal, vigilance and narrowing of attention, in addition to supporting processes to activate the HPA axis.^10^ Moreover, during an acute stressor, the immune system initiates a cascade of defensive responses to enhance immunoprotection.^12^ Specific cells are activated and enter the bloodstream, potentially preparing the body for injury or infection during the fight‐or‐flight response. Additionally, acute stress also increases the levels of pro‐inflammatory cytokines in the blood.^13^

These physiological responses are intricately connected to the production of endogenous GCs, such as the stress hormone cortisol, regulated by the HPA axis. When induced, the hypothalamic corticotropin‐releasing hormone (CRH; released by the hypothalamic paraventricular nucleus (PVN)) stimulates the pituitary gland to release adrenocorticotropic hormone (ACTH). In return, ACTH stimulates the production and secretion of GCs by the adrenal cortex.^14^ CRH release from the PVN is inhibited by GCs via membrane GRs, thereby reducing GC production via negative feedback mechanisms. The termination of CRH secretion prompts a decline in ACTH release, leading to a gradual restoration of GC secretory activity to its baseline level.^15^ Upon activation of the HPA axis, increased GC levels facilitate fuel availability by enhancing white adipose tissue lipolysis, liberating fatty acids as energy source.^16^ Furthermore, GCs promote gluconeogenesis in the liver and decrease glucose uptake in skeletal muscle and white adipose tissue to preserve plasma glucose for the brain during stress and promote maximal brain function.^17^ Once the perceived danger dissipates or the stressor is resolved, the body returns to its normal homeostatic state and GC levels decline.

When managed appropriately, acute stress generally does not lead to long‐term health problems. When acute stressors are more frequent and/or severe, they can lead to chronic stress responses and their associated health risks.

### 2.2 Chronic stress

When a stressor exceeds a temporal threshold in duration or severity, the adaptive stress responses can become, in part, maladaptive, leading to adverse effects, such as anxiety and cognitive dysfunction, mood alteration, immune dysfunction and adverse metabolic effects including central obesity.^12^, ^18^, ^19^ Chronic stress is often the result of prolonged exposure to stressors, e.g., caused by work‐related issues, health issues or financial problems. Consequently, chronic stress is characterized by its persistence over an extended period of time, where it can last for weeks, months or even years. While the initial mechanism of the chronic stress response is similar to that of the acute stress response, the persistent activation of the stress systems can lead to more harmful effects on the body through various mechanisms. For example, chronic stress can lead to long‐term elevation in GC levels, exemplified by cortisol, leading to a state of chronic stress system activation, which can result in various psychological and metabolic effects,^10^ which are discussed in the following paragraph. On the other hand, evidence also suggests that extreme cases of long‐term chronic stress can eventually lead to reduced cortisol reactivity through inhibition of the HPA axis and adrenal hypoactivity,^20^ highlighting the complex interplay between chronic stress and HPA axis functioning.

## 3 STRESS AND THE CONNECTION WITH OBESITY

Obesity is a chronic relapsing disease that is associated with the development of various comorbidities, thereby resulting in increased morbidity and mortality.^21^ Obesity rates are rapidly increasing, with major consequences for public health. A recent study published by the Lancet has shown that, in 2022, more than 1 billion people were living with obesity. They also report that 43% of the adults were living with overweight in 2022.^22^ The World Obesity Federation expects the number of people living with overweight or obesity to increase to 51% in 2035.^23^ While obesity is often viewed through the lens of excessive caloric intake and physical inactivity, the aetiology of obesity is far more complex. There are several underlying factors that can contribute to the development of obesity or maintenance of obesity. Causes can be divided into two: (1) societal causes, as the obesogenic environment with respect to exposure to ultra‐processed, unhealthy food, technology diminishing the need to move in daily life and endocrine disrupting chemicals in many products we use, and also (2) individual's causes. The latter comprise lifestyle factors, mental and social factors, genetics, certain medications and endocrine or hypothalamic diseases.^24^ The key developmental hallmarks in obesity are expansion and low‐grade inflammation of the adipose tissue. Initially, a positive energy balance leads to an increase in triglyceride storage within adipocytes, causing these cells to enlarge, also known as hypertrophy. Another mechanism to enhance adipose tissue storage capacity is formation of new adipocytes through hyperplasia. This expansion of adipose tissue is a key feature of obesity.^25^ In the past years, there is increasing evidence for a relation between chronic stress and the development of obesity.^3^, ^26^ Indeed, a meta‐analysis showed a significant positive association between perceived stress (assessed by stress scales) and BMI, waist circumference and serum triglyceride levels.^27^ Furthermore, higher levels of perceived stress have been associated with lower levels of eating awareness (with greater consumption of fast food meals), physical activity and walking.^28^ Moreover, as described in more detail below, chronic stress can also lead to immune cell dysregulation causing low‐grade inflammation, which is another hallmark of obesity.^29^ This is characterized by an increase in the number of macrophages in adipose tissue, which shift from an anti‐inflammatory M2 phenotype to a pro‐inflammatory M1 phenotype. This shift results in the secretion of pro‐inflammatory cytokines, such as tumour necrosis factor‐alpha (TNF‐α), interleukin‐6 (IL‐6) and IL‐1 receptor antagonist (IL‐1Ra), which contribute to low‐grade systemic inflammation and metabolic dysregulation.^30^ Additionally, stress‐induced obesity can alter the function of other immune cells, including T cells, further promoting an inflammatory state and disrupting normal adipose tissue metabolism.^31^ Stress is acknowledged more and more as a potential contributor to the development of obesity and subsequent cardiometabolic diseases and the assessment of stress is even mentioned in the clinical work‐up diagnostics of obesity.^24^, ^32^

However, next to ongoing stressors that contribute to chronic stress, including living in poverty and work stress, there are also milder but more frequent daily stressors that exist. These ‘daily hassles’, or daily annoyances, include concerns about weight, being lonely, or momentary stressors such as due to balancing a busy work and family life. Of note, daily hassles might be minor in comparison with life events, e.g., losing a loved one, but are also associated with negative effects on mental and physical health and appear to exacerbate already existing physical health conditions.^33^, ^34^ However, whether these daily hassles also have long‐term implications for cardiometabolic health including obesity, is largely unknown. It has been proposed that continuous physiological alterations may result in biological wear‐and‐tear, potentially heightening an individual's vulnerability to illness later in life.^34^

When looking at the relation between stress and obesity, an often overlooked aspect is that obesity on itself can also induce stress. Almost all over the world, a strong weight stigma exists. Weight stigma is characterized by prejudiced, stereotyped and discriminatory views and actions targeted towards individuals because of their weight and size and is highly prevalent among individuals living with overweight or obesity.^35^, ^36^ Despite this, evidence on obesity stigmatization remains abundant throughout areas of media and the political and public health landscape.^37^ This profound stigma has negative consequences on mental and physical health outcomes and further contributes to the development of obesity.^38^, ^39^ In this way, a vicious circle may be formed between stress and obesity, leading to more weight gain and poorer mental and physical health among individuals living with overweight or obesity.^15^, ^40^ This effect may be intensified by the use of exogenous GCs prescribed for coexisting conditions associated with obesity, such as osteoarthritis and asthma. In prior studies we have reported significantly higher use of corticosteroid‐containing medications among individuals with obesity.^41^ In addition, also biological mechanisms exist by which obesity can further contribute to biological stress. More specifically, obesity is associated with tissue‐specific alterations in GC metabolism. In cross‐sectional studies it has been shown that in patients with obesity, more cortisol is generated within white adipose tissue by increased activity of 11β‐hydroxysteroid dehydrogenase type 1 (11β‐HSD1), which may play part in the maintenance and potentially aggravation of obesity and associated cardiometabolic diseases.^42^

Taken together, chronic stress is associated with detrimental effects on both mental and physical health. This includes heightened vulnerability to anxiety and depression as well as an increased risk of central obesity, type 2 diabetes and cardiovascular disease.^43^ GCs are bidirectional related to abdominal obesity. The latter may be further exacerbated by the obesity stigma, exogenous GC use and tissue‐specific GC production. These stress‐related conditions are a massive public health burden, as a result of which the WHO has dubbed stress the ‘Health Epidemic of the 21st Century’.

**FIGURE 3:**
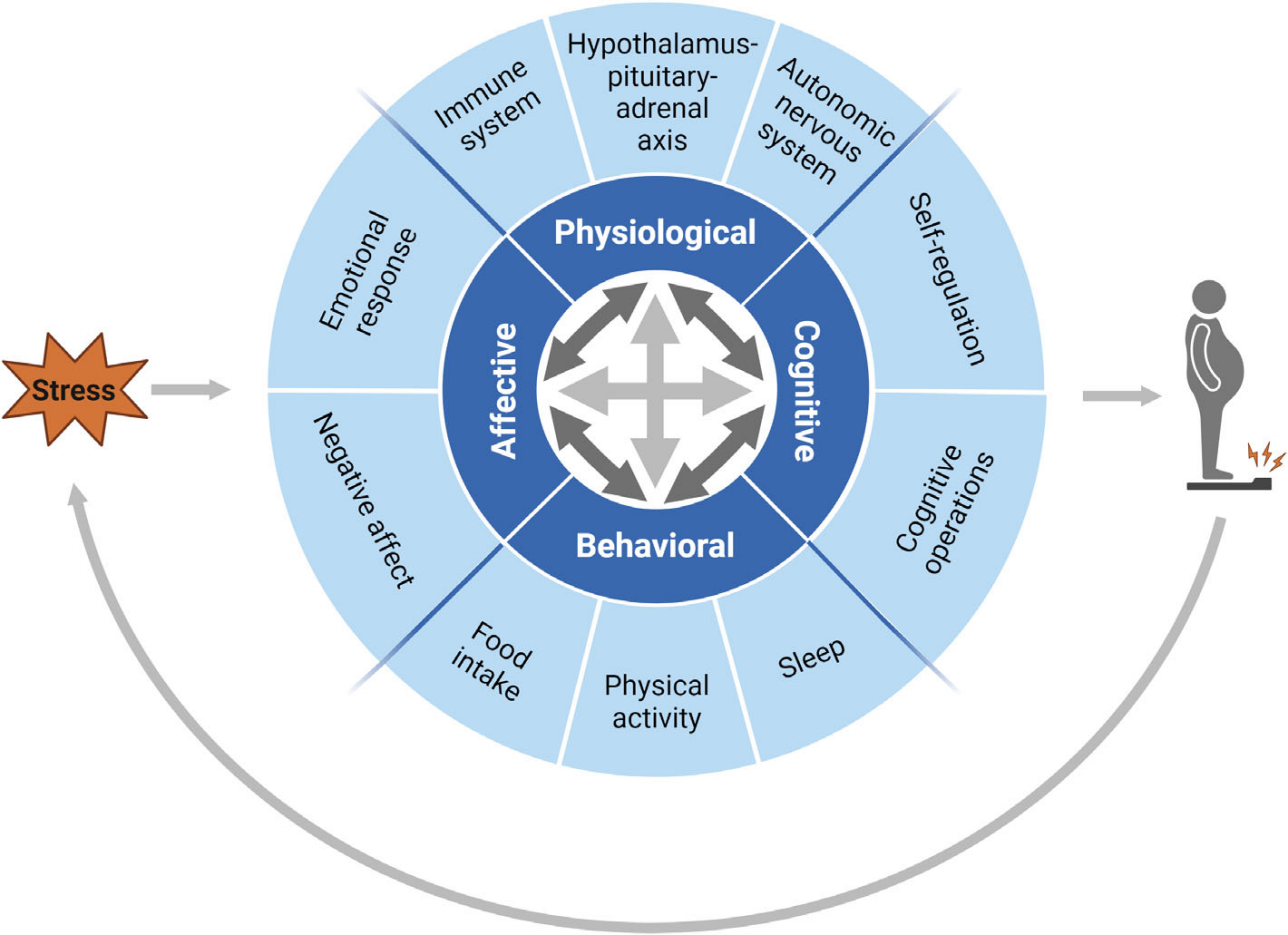
Stress response pathways describing the stress–obesity connection, highlighting the significance of understanding stress across the physiological, affective, cognitive and behavioural domain. Weight stigma, exogenous glucocorticoid use and tissue‐specific glucocorticoid production can subsequently increase stress, thus creating a vicious circle with negative consequences on mental and physical health outcomes. *Source*: Created with BioRender.com.

To delve deeper into the stress–obesity connection, it is important to understand that exposure to stress can generate a wide range of observable stress responses in different domains. More specifically, stress responses can occur in the physiological, affective, cognitive and behavioural domains. In this review, we particularly focus on the physiological domain of the stress response, specifically the stress hormone cortisol. The four domains of the stress response can interact with each other over time, creating dynamic changes within an individual (Figure 3).^44^ Understanding these domains provides a more profound perspective on how stress contributes to the development of obesity.

## 4 PHYSIOLOGICAL STRESS RESPONSE

### 4.1 Glucocorticoid exposure and obesity

Chronic stress results in long‐term increases in the GC hormone cortisol. Chronically increased GC levels, exemplified by endogenous Cushing's syndrome or mimicked when using high dosages of exogenous synthetic GCs, are causally related with the development of obesity, metabolic syndrome and eventually cardiovascular disease.^45^ However, an important question is whether also more subtle chronic increases in cortisol levels, such as in case of chronic stress, are linked to obesity and cardiometabolic diseases.

To understand the link between enhanced GC levels and body weight, more detailed information on the role of cortisol in adipose tissue metabolism is needed. Cortisol is primarily bound to corticosteroid‐binding globulin (CBG) when transported in the bloodstream. Approximately 90% of circulating cortisol forms complexes with CBG, while about 6% binds with a lower affinity to albumin. Thus, only around 4% of cortisol remains free and biologically active.^46^ Cortisol has a circadian rhythm which shows low levels at the initial part of sleep, a steep rise in the late night, and a peak within 30–45 min after awakening. Cortisol exerts its effects in target tissues upon binding to the GR or mineralocorticoid receptor (MR), which are both nuclear receptors. More specifically, binding of cortisol to the GR or MR initiates a cascade of molecular events that modulate the expression of multiple target genes, influencing various physiological processes, including glucose and lipid metabolism, immune function and the body's response to stress.^18^ The affinity of cortisol to the GR is about 10‐fold lower compared to the MR, resulting in partial occupancy of the GR when cortisol levels are low. Therefore, the MR experiences significant occupancy even during basal states (late evening), whereas during stress levels or the circadian peak of cortisol secretion (morning), the GR is gradually stimulated.^47^ GRs are present in cells in the hypothalamus and pituitary gland, which are part of the negative feedback regulation of cortisol production.^48^ Besides, GRs can also be found within neurons in various other regions of the brain, including the hippocampus, frontal cortex and amygdala. The expression and functionality of the GR is crucial for mediating the effects of GCs on stress responses and mood regulation.^49^, ^50^ Cortisone is the biologically inactive form of cortisol. The ratio of cortisone and cortisol is tissue specific and is regulated by the expression levels and activity of the 11β‐HSD enzymes. Specifically, 11β‐HSD1 has the capability to (locally) convert cortisone to the biologically active cortisol, whereas 11β‐HSD2 serves to inactivate cortisol and locally control the action of GCs in particular tissues, e.g., in the kidney.^46^

While cortisol serves many roles in the body aiming at mobilizing energy to support the stress response, on the longer term, it can also trigger processes that lead to weight gain. For instance, various psychoneuroendocrine models on stress and obesity have described interactions between the HPA axis with various appetite‐regulating hormones.^51^ Herein, an important difference exists between acute and chronic stress. Acute stress can cause suppression of appetite via CRH release, leading to an inhibition of orexigenic NPY/AgRP neurons.^52^ In contrast, chronic stress, by chronically increased levels of GCs, can induce an increase in food intake and a preference for high caloric foods. This latter effect is suggested to be partly mediated by GC‐induced alterations in the levels and/or signalling of appetite‐regulating hormones, such as lowering of the satiety hormone leptin and increase of the hunger hormone ghrelin.^51^ Furthermore, increased GC levels can stimulate the expansion of undesirable visceral adipose tissue stores, by relocation of fat storage from peripheral subcutaneous adipose tissue (e.g., arms and legs) to visceral adipose tissue, while also hampering the beneficial functionality of brown adipose tissue.^18^ This particular distribution and the privileged effects of GCs in the visceral adipose tissue, might partially be explained by the greater density of GRs in this location as compared with other fat depots.^4^ In addition, GCs can also increase the expression levels of certain genes involved in fat deposition.^53^ Moreover, there is increasing evidence that emphasizes the importance of circadian variations in GCs in relation to meal timing in influencing their anabolic effects on adipocytes.^53^

### 4.2 Variations in glucocorticoid levels

Findings regarding the relationship between cortisol levels as measured in blood, saliva and urine, and obesity show inconsistencies. These may in part be attributed to variations in the diurnal cortisol secretion pattern.^54^ The connection between long‐term cortisol levels measured in scalp hair with BMI appears to exhibit stronger consistency across diverse populations. Long‐term cortisol and cortisone exposure can be assessed retrospectively by measuring these factors in hair using liquid chromatography–tandem mass spectrometry (LC–MS/MS).^55^ Since hair typically grows at a rate of about 1 cm/month, hair analysis provides the opportunity to depict the average long‐term systemic cortisol levels, potentially reflecting functioning of the HPA axis.^56^ Interestingly, it has been demonstrated that, on average, individuals with obesity have higher cortisol levels as measured in scalp hair compared to individuals living with overweight or normal weight.^57^ Our meta‐analysis investigating GC levels measured in scalp hair in relation to anthropometrics found a positive association between hair cortisol and BMI.^26^ This relation was even stronger when looking at hair cortisone levels, the inactive form of cortisol, in association with waist circumference. In addition, increased hair cortisol levels have also been associated with increases in BMI and waist circumference in longitudinal analyses.^3^ Moreover, in our recent study, we observed that increased hair cortisone levels were associated with incident cardiovascular disease after 5–7 years of follow‐up, in particular, in young individuals.^58^

Thus, these findings highlight a robust correlation between long‐term cortisol exposure and both overall body mass and central adiposity, indicating that elevated cortisol levels caused by chronic stress are associated with the development of obesity.

### 4.3 Variations in glucocorticoid rhythmicity

As described above, cortisol has a circadian rhythm. The disruption of cortisol rhythms is linked to diverse impacts on metabolism, behaviour and cognition, along with its association with endocrine disorders such as Cushing's syndrome and primary aldosteronism.^59^, ^60^, ^61^ In a cohort of Europids, it has been shown that alterations in diurnal cortisol patterns were predictive of future glucose disturbances.^62^ In addition, disrupted diurnal salivary cortisol rhythms have been associated with obesity. A study performed in White, Hispanic and Black men and women showed that both BMI and waist circumference were negatively correlated with awakening cortisol and positively correlated with the early decline slope. This suggests that higher BMI and waist circumference are associated with neuroendocrine dysregulation.^63^ Recently, a microdialysis device has been developed for minimally invasive and comprehensive monitoring of diurnal fluctuations in adrenal steroid hormones.^64^ Samples can be collected through a catheter inserted under de abdominal skin, which allows to gather interstitial fluid samples every 10–20 min over 24–72 h for subsequent analysis via LC–MS/MS. This technique has the ability to measure free cortisol levels with high measurement frequency and holds the potential to advance our understanding of hormone dynamics in the context of stress, obesity and other diseases.

### 4.4 Variations in glucocorticoid sensitivity

Interestingly, in a previous study, it was discovered that roughly half of the individuals with obesity exhibited high hair cortisol levels, while the other half exhibited normal hair cortisol levels, as compared to individuals living with overweight and normal‐weight individuals.^57^ It is not known why some persons with obesity have high systemic cortisol levels, and others not, and whether the latter group may be more sensitive to the effects of GCs. It has been shown that the biological effects of cortisol are determined not only by its quantity, but also by the extent to which it can signal via the GR in target tissues, including the adipose tissue depots in order to exert e.g. metabolic or anti‐inflammatory effects, so‐called GC sensitivity. Of note, individual differences in GC sensitivity in different tissues exist.^18^

The GR acts as a key mediator in the stress response and—as mentioned above—exerts its effect in target tissues by regulating the expression of specific genes, the so‐called GR‐target genes.^65^ These genes can regulate various processes in tissues, including lipid storage in adipose tissue. Importantly, certain GR polymorphisms that are associated with increased GC sensitivity (e.g., N363S, BclI) have been shown to be linked to more fat mass and increased risk to develop type 2 diabetes.^66^ In addition, increases in GR expression levels and activity have been linked to diabetes pathogenesis in mice and rats, and linked to the metabolic syndrome in human skeletal muscle.^67^ In contrast, GR polymorphisms that are associated with relative GR resistance (e.g., ER22/23EK, 9ß) are associated with less central obesity, a beneficial lipid profile, and sex‐specific body composition with more lean mass and greater muscle strength in men, and smaller waist circumference in women.^18^, ^66^

Several *in vivo* and *in vitro* methods are used to study GC sensitivity. An *in vivo* method is the dexamethasone (a synthetic GC) suppression test which is often used in the diagnostic work‐up for Cushing's syndrome to assess the presence of autonomous cortisol production. However, in the absence of autonomous cortisol production, this method can also be used to assess GC sensitivity based on the suppression of cortisol following dexamethasone. In most people, the conventional 1 mg of dexamethasone fully suppresses the HPA axis. However, if persons are relatively insensitive to 1 mg dexamethasone due to, e.g., lower GR activity, the suppression will be less, as has been observed in carriers of the ER22/23EK polymorphism of the GR gene. This polymorphism has indeed been shown to be linked to a more inactive GR, which may explain the relative GR resistance.^68^ In addition, to measure variation across the continuum of the HPA axis reactivity, as a proxy of GC sensitivity, a variant of this test employing a very low dose of dexamethasone (0.25 mg) can be used.^69^ If persons fully suppress cortisol levels with this test, it indicates increased sensitivity to GCs. It should be noted that this test may not fully represent local (acquired) GC sensitivity since there is substantial evidence that GC sensitivity can differ among tissues, which is also influenced by genetic and environmental factors.^70^

Several *in vitro* assays have been developed to assess GC sensitivity in different target tissues such as in peripheral blood mononuclear cells (PBMCs). One of these assays measures the GR‐mediated transactivation or transrepression of four well‐defined GC‐responsive genes: GILZ, FK506 binding protein 5 (FKBP5), IL‐2 and IL‐6, in response to increasing dosages of dexamethasone using reverse transcription‐quantitative polymerase chain reaction (RT‐qPCR).^71^ FKBP5 together with heat shock proteins 90 and 70 forms a chaperone complex, regulating GC sensitivity. Upon binding of GCs to the GR, the GR undergoes conformational changes, leading to its release from the chaperone complex and subsequent translocation into the nucleus. Inside the nucleus, the GR–ligand complex can bind to GC response elements (GREs) and interact with other transcription factors, thereby regulating the expression of various target genes.^18^ Genetic and epigenetic variations in FKBP5 are linked to altered stress reactivity and increased risk for psychiatric disorders.^72^ Moreover, transactivation by the GR via GREs is thought to play a major role in most of the unfavourable metabolic effects of GCs.^73^ Since this assay is labour‐intensive, it is not yet possible to incorporate this method in routine clinical application. To what extent the measured GC sensitivity in PBMCs represents GC sensitivity in all organs and in metabolism, and what the exact role is of the GR and GC sensitivity in the link between stress and obesity development, remains to be determined.

### 4.5 Other physiological stress responses

Besides the HPA axis, also parts of the ANS—specifically the SNS—partake in the physiological stress response and appear altered in individuals living with obesity. The ANS has two divisions, the SNS and the parasympathetic nervous system (PNS). Stressors can induce an increase in SNS activity and decrease in PNS activity, which can be observed in various systems such as the heart (e.g., rate, stroke volume), respiration and blood vessels resulting in vasoconstriction and vasodilation in various organs.^74^ Several studies in literature propose that the ANS of individuals with obesity is chronically altered. Given the role of the ANS in energy metabolism and cardiovascular regulation, alterations in the ANS can significantly contribute not only to the development of obesity but also the pathophysiology of diverse cardiac complications.^75^ The ANS has a central communication role between the gastrointestinal system and CNS for energy homeostasis. For instance, the ANS influences energy intake by inducing satiety after a meal via the vagal nerve, which is activated after gastric distension and the release of gut hormones such as cholecystokinin, peptide Y, glucagon‐like peptide‐1, ghrelin and leptin. These hormones can inhibit gastric emptying, improve food intake control and may also increase energy expenditure.^5^ However, in individuals living with obesity, these metabolic signalling pathways often become impaired, leading to dysregulated brain circuits involved in metabolic regulation, and resulting in compensatory but ineffective over activation of the SNS.^5^ Increased plasma leptin levels, metabolic abnormalities such as insulin resistance and glucose intolerance, and baroreflex dysfunction all contribute to increased SNS activity. Although SNS activation typically increases energy expenditure, in obesity, this effect is diminished due to β‐adrenergic downregulation which may hamper the SNS‐induced increase in energy expenditure. Simultaneously, selective leptin‐resistance further disrupts energy balance by impairing satiety. These metabolic disturbances can contribute to the pathophysiological changes related to obesity development, as well as hypertension, and renal and cardiovascular complications.^5^, ^76^, ^77^ Therefore, despite SNS activation, the expected increases in energy expenditure and proper satiety signalling are often compromised, contributing to the challenges in managing obesity and its associated health risks.

The HPA axis is also interrelated with the immune system, the third stress system, through a network of cellular and humoral signals, including cytokines. As obesity is characterized by increased GC levels and chronic low‐grade inflammation, it is likely that there is an imbalance in the interaction between HPA axis and the immune system.^3^ Chronic stress can disrupt the balance of immune function, leading to immune dysregulation characterized by heightened inflammation and altered immune cell activity. This can in turn influence adipose tissue biology, contributing to adipocyte dysfunction and accumulation of visceral fat.^12^, ^29^ It has been shown that adipose tissue in individuals living with obesity, secretes high levels of pro‐inflammatory adipokines such as leptin, visfatin and resistin.^78^ In addition, inflammation can contribute to the development of insulin resistance in several tissues, including skeletal muscle, whereby visceral adipose tissue is an important determinant in insulin resistance progression due to its role as a pro‐inflammatory depot.^12^, ^29^, ^78^ Chronic inflammation, next to long‐term stress, has been associated with increased risk of various diseases, such as infectious illnesses, cardiovascular disease, diabetes, obesity, certain cancers and autoimmune disease, as well as a decline in overall health and higher mortality rates.^3^, ^7^ The link between chronic stress and inflammation in the onset of certain diseases may be explained by the development of GC resistance due to prolonged exposure to stressors. This can in turn cause dysregulated HPA axis functioning and can impact the appropriate regulation of inflammation.^7^ Besides the low‐grade inflammation characteristics of obesity being related to metabolic abnormalities, associations have also been found between inflammatory markers and obesity‐related eating behaviours. Several studies have observed a positive relation between C‐reactive protein (CRP)/high‐sensitivity CRP and loss of control in eating. Current evidence suggests a bidirectional relationship between inflammatory/immune markers and obesity‐related eating behaviours, indicating that inflammation may both influence and be influenced by eating behaviours associated with obesity.^79^ Taken together, the intricate crosstalk between stress and immune dysregulation forms a complex network of interactions contributing to obesity pathogenesis.

## 5 ADDITIONAL DOMAINS OF THE STRESS RESPONSE IN RELATION TO OBESITY

### 5.1 Affective domain

Part of the pathways that lead from stress to obesity are an individual's effort to manage the adverse emotional facets of stress. Daily life events and stressful situations can lead to subjective (negative) emotional feelings, such as lowered mood, fear and anxiety. Theories explain that emotional well‐being is largely dependent on how people respond to adverse life events; whether it was unexpected, controllable, can be coped with, was one's own fault, and so on.^44^ Studies have shown that higher levels of negative affect are associated with increased odds of having obesity.^80^, ^81^ It is suggested that this association might be mediated by emotional eating and maladaptive coping strategies.^80^ In controlled laboratory conditions, emotional distress can lead to increased consumption of ‘comfort’ foods in both humans and animals, even when they are not experiencing hunger and have no physiological requirement for additional calories.^82^ Moreover, daily hassles and depressed mood have been shown to be associated with unhealthy eating behaviour, which might thus be a pathway by which stress can influence obesity development and health in general.^83^, ^84^ Contrary to the observed evidence for an association between negative affect and obesity, positive affect does not seem to influence the likelihood of having obesity.^80^, ^81^ Findings suggest that the average level of negative effect that people experience and how they emotionally respond to daily hassles have long‐term implications for mental health as well.^85^ In addition, emotional distress can also trigger physiological responses in the body, including the release of cortisol and inflammatory mediators such as cytokines. Persistent activation of the HPA axis and immune response can disrupt metabolic processes, leading to weight gain and the development of obesity.^29^ Exploring the emotional domain of the stress response allows us to better understand stress‐related emotional feelings and how these contribute to unhealthy eating behaviour, HPA axis activation and inflammation, as significant factors in obesity development.

Apart from these adverse effects being consequences of stress, poor health in itself can also reduce resilience and increase vulnerability to new stress exposures. This can lead to a downward spiralling process, leading to decreased health and in turn reduces the threshold at which events are perceived as threatening.

### 5.2 Cognitive domain

Stress can affect hot and cold cognitive function in many ways. Hot cognition refers to decision‐making based on emotions, while cold cognition involves more rational, or logic‐based thinking.^86^ In fact, almost all hot and cold cognitive operations are susceptible to being affected by stress.^87^ Chronic stress often triggers emotional eating, where individuals turn to consumption of ‘comfort’ foods, driven by hot cognition.^82^ Furthermore, stress can affect cold cognitions, such as self‐regulation, important for controlling one's behaviour and thereby relevant to enact obesity‐preventing behaviour such as eating and physical activity.^88^ Stress can undermine self‐regulatory cognitive processes and interfere with brain areas responsible for self‐regulation.^89^ The interaction of GCs and adrenergic systems in specific brain regions is hypothesized to be an important mediating mechanism in the actions displayed by stress on cognition.^87^ Consequently, by hindering cognitive processes such as self‐regulation, stress can contribute to development of obesity. Investigating the cognitive domain can help in identifying cognitive processes involved in weight gain, and guide interventions aimed at improving stress management.

### 5.3 Behavioural domain

Several aspects of human behaviour, such as the fight‐or‐flight response (active conflict/social withdrawal), are part of the stress response. Investigating the behavioural domain helps us understand the behavioural mechanisms linking stress and obesity, including, among others, eating, physical activity and sleeping.

Stress‐related eating as well as the consumption of foods high in fat and sugar can be a means of coping with stressful experiences.^90^ It has been shown that the presence of daily hassles, such as ego‐threatening, interpersonal and work‐related hassles, were associated with a reduction in consumption of main meals and vegetables and increased consumption of high fat/sugar snacks.^83^ Furthermore, individuals who are more reactive to stressors—shown by increased levels of saliva cortisol after a stress test (afternoon)—are more likely to engage in stress‐related eating.^91^ Whether stress‐related eating might actually partially mediate the association between stress and obesity needs to be further studied, as this knowledge would allow for further optimization of evidence‐based treatments addressing obesity and weight management.

Stress can also disrupt activity patterns, either by increasing sedentary behaviour and/or by decreasing physical activity.^89^ A study among 1382 women showed longitudinal associations between perceived stress and less leisure‐time physical activity, as well as with increased television viewing time.^92^ Furthermore, a comprehensive systematic review, including 168 studies, concluded that the experience of stress impairs efforts to be physically active.^93^ To summarize, evidence shows that stress influences eating behaviour and disrupts activity patterns, which can in return lead to a higher likelihood of obesity.

Besides influencing eating behaviour and disrupting activity patterns, stress can also influence sleep quality and duration, and vice versa, sleep deprivation has been shown to be a contributor to heightened stress levels. Sleep deprivation is associated with elevated cortisol levels and increased cortisol responses to stressors, which can be indicative of elevated HPA axis response.^94^ Short sleep duration (<6 h) has been shown to play a role in the risk of future obesity.^95^ Shorter sleep can promote fatigue, reduce physical activity, increase sedentary behaviour and increase hunger (particularly for high‐fat and carbohydrate foods),^89^, ^96^, ^97^ which might be mediated by elevated cortisol levels. A randomized controlled pilot study showed that extending sleep in adult short sleepers (5 to <7 h) reduced free sugar intake, consequently improving diet quality.^98^ Increasing sleep duration could be a practical approach to help control the excessive consumption of free sugars in an obesity‐promoting environment.

There are many pathways that tie sleep deprivation to obesity, but more research is needed to the bidirectional relationship between stress and sleep deprivation, and the complex interplay with the development of obesity.

## 6 STRESS AND OBESITY: TOWARDS NOVEL TREATMENT MODALITIES

Addressing stress‐related factors in the treatment of obesity presents a multifaceted challenge that requires a comprehensive approach, including lifestyle modifications, behavioural interventions, psychosocial support and pharmacological interventions. Several potential treatment targets have emerged in the intersection of stress and obesity, aiming to mitigate the adverse effects of chronic stress on weight regulation and metabolic health.

Behavioural interventions often focus on stress management techniques, such as mindfulness‐based stress reduction (MBSR) and cognitive–behavioural therapy (CBT). MBSR has been shown to significantly reduce stress and fasting glucose levels in women living with overweight or obesity.^99^ In addition, CBT interventions can be used to implement lifestyle changes including regular physical activity, proper nutrition, quality sleep and stress management, especially for weight loss and weight maintenance.^100^ Accordingly, we recently showed that using this combined approach (including CBT, nutritional advice and physical activity) in patients with obesity was associated with significant improvements in body weight and body composition, accompanied with improvements in cardiometabolic, endocrine, psychological and behavioural parameters. In addition, we also observed significant decreases in the inflammatory markers sIL‐2R, IL‐1ra, VEGF and sMR, which can be expected to have favourable effects on cardiometabolic health and various other diseases.^101^ Furthermore, psychosocial support, such as peer support groups aimed to provide informational, appraisal and emotional assistance, is shown to decrease weight and BMI in individuals living with overweight or obesity and might also have positive effects on reducing perceived stress levels by improving social support.^102^

Pharmacotherapy can complement behavioural interventions and lifestyle modifications, by targeting stress‐related pathways which offer potential options for managing obesity. Drugs, such as GR antagonists or 11β‐HSD1 inhibitors, the latter reducing the synthesis of cortisol, have shown promise, however until now only in preclinical studies.^67^ General and hepatic inactivation of the GR, through the use of antagonists, has been shown to improve glucose tolerance and insulin resistance in animals with diabetes.^67^, ^103^ To date, mifepristone is the only clinically approved GR antagonist. As this GR antagonist also exhibits cross‐reactivity with other nuclear steroid receptors, more selective GR antagonists are currently investigated.^104^ Furthermore, in hyperglycaemic mouse models, the use of specific 11β‐HSD1 inhibitors has demonstrated the ability to decrease glucose intolerance, lowering food intake and weight gain.^67^ Translation of this research on 11β‐HSD1 inhibitors from preclinical studies has proved challenging so far, with studies not meeting their primary endpoints.^105^ However, it does remain a promising area, whereby more research is needed to discover the therapeutic potential of 11β‐HSD1 inhibitors and potentially target specific persons who have a stress‐related type of obesity.

Integrated treatment approaches that combine all of the above may hold promise for addressing the complex interplay between stress and obesity while addressing individualized patient needs and preferences.

## 7 CONCLUDING REMARKS

The stress–obesity connection consists of an intricate and multifaceted relationship which has big impact on public health. Chronic exposure to stress has been identified as a potential contributor to the development of obesity and associated cardiometabolic diseases. In turn, obesity itself can also contribute to the activation of the stress systems.

This review has highlighted the significance of understanding stress responses across the physiological, affective, cognitive and behavioural domains, and the interactions between them. The physiological impact of stress on adipose tissue distribution and metabolic function, mediated by cortisol and influenced by GC sensitivity, underscores the complexity of this interplay. Furthermore, the connections between the HPA axis, ANS, immune system and behavioural factors are crucial for a holistic understanding of how stress influences obesity. The affective and cognitive dimensions shed light on the psychological aspects influencing eating behaviour and self‐regulation, while behavioural mechanisms—including, among others, eating, physical activity and sleeping—contribute to the multifaceted nature of this connection. To effectively address the stress–obesity connection, a comprehensive approach that considers these multi‐level interactions, is thus essential. Potential treatment strategies that impact on stress, including lifestyle modifications, behavioural interventions, psychosocial support and pharmacological interventions, should therefore be integrated with an understanding of how these also interact with the physiological stress systems.

Collaborations between researchers and healthcare professionals are essential to bridge the existing gaps in stress research. By synthesizing knowledge across various disciplines, we can enhance our understanding of the connections between stress and obesity. A comprehensive approach is crucial, not only for understanding the link between stress and obesity, but also for more effective obesity prevention and management strategies with an emphasis on reduction of the impact of stress.

## AUTHOR CONTRIBUTIONS

*Conceptualization*: R. L., M. S., M. B. and E. R. *Literature search and generation of figures*: R. L., M. S. and M. B. *Funding acquisition*: B. P. and E. R. *Supervision or mentorship*: M. B. and E. R. Each author contributed important intellectual content during manuscript drafting or revision and had final approval of the submitted and published versions.

## CONFLICT OF INTEREST STATEMENT

All authors declare that they have no relevant financial interests or disclosures to report.
